# Differential Role of p53 in Oligodendrocyte Survival in Response to Various Stresses: Experimental Autoimmune Encephalomyelitis, Cuprizone Intoxication or White Matter Stroke

**DOI:** 10.3390/ijms222312811

**Published:** 2021-11-26

**Authors:** Fucheng Luo, Zhen Zhang, Yu Luo

**Affiliations:** Department of Molecular Genetics and Biochemistry, College of Medicine, University of Cincinnati, Cincinnati, OH 45214, USA; luofc@lpbr.cn (F.L.); 15559780381@163.com (Z.Z.)

**Keywords:** p53, oligodendrocyte, inflammation, cuprizone, white matter stroke

## Abstract

Promoting oligodendrocyte viability has been proposed as a therapeutic strategy for alleviating many neuronal diseases, such as multiple sclerosis and stroke. However, molecular pathways critical for oligodendrocyte survival under various stresses are still not well known. p53 is a strong tumor suppressor and regulates cell cycle, DNA repair and cell death. Our previous studies have shown that p53 plays an important role in promoting neuronal survival after insults, but its specific role in oligodendrocyte survival is not known. Here, we constructed the mice with oligodendrocyte-specific p53 loss by crossing TRP53^flox/flox^ mice and CNP-cre mice, and found that p53 was dispensable for oligodendrocyte differentiation and myelin formation under physiological condition. In the experimental autoimmune encephalomyelitis (EAE) model, p53 loss of function, specifically in oligodendrocytes, did not affect the EAE disease severity and had no effect on demyelination in the spinal cord of the mice. Interestingly, p53 deficiency in oligodendrocytes significantly attenuated the demyelination of corpus callosum and alleviated the functional impairment of motor coordination and spatial memory in the cuprizone demyelination model. Moreover, the oligodendrocyte-specific loss of p53 provided protection against subcortical white matter damage and mitigated recognition memory impairment in mice in the white matter stroke model. These results suggest that p53 plays different roles in the brain and spinal cord or in response to various stresses. Thus, p53 may be a therapeutic target for oligodendrocyte prevention in specific brain injuries, such as white matter stroke and multiple sclerosis.

## 1. Introduction

Oligodendrocytes (OLs) are the myelinating glial cells in the central nervous system (CNS). They produce myelin to wrap around axons for ensuring rapid action potential conduction along the axon. OLs are also involved in neuroplasticity and provide trophic support to axonal and neuronal maintenance. Due to their high plasma membrane specialization and metabolic rate, OLs are highly vulnerable to various insults, such as ischemic inflammation, oxidative stress and excessive excitatory neurotransmitter [1]. Cell death, apoptosis and necrosis of OLs and subsequent demyelination have been considered as some of the mechanisms underlying many neurological disorders, such as ischemic stroke, multiple sclerosis (MS), Alzheimer’s disease and amyotrophic lateral sclerosis. In addition to enhancing OL differentiation and myelin repair, experimental studies demonstrate that the protective strategy of OLs and myelin can provide complementary effects in alleviating disease progression [2]. However, the molecular mechanisms that trigger OL damage and demyelination in these neurological disorders are still unclear.

The tumor suppressor p53 is a well-known stress response gene and can regulate the process of apoptosis, necroptosis and ferroptosis. Accumulating evidence indicates a mechanistic link between p53 and the pathogenesis of neurological disorders. Genetic deletion or pharmacological inhibition of p53 can provide protection against neuronal damage. Our previous studies also demonstrated that dopaminergic neuron-specific deletion of p53 gene can attenuate methamphetamine or 1-methyl-4-phenyl-1,2,3,6-tetrahydropyridine (MPTP)-induced neurotoxicity [3]. Forebrain neuronal specific ablation of the p53 gene provides protection in a cortical ischemic stroke model [4]. In addition to neuron, p53 is also involved in OL survival in response to various stresses. p53 contributes to immune-mediated cell death of human and rodent OLs in vitro [5,6]. p53 mediates oligodendrocyte apoptosis by enhancing ER–mitochondria interaction and by triggering the E2F1-mediated apoptosis pathway after compressed spinal cord injury [7]. In cuprizone-induced demyelination model, the global knockout and pharmacological inhibition of p53 decreased the extent of oligodendrocytic apoptosis, demyelination and microglial recruitment in the corpus callosum [8]. However, it is still unclear whether the effect reported in these previous studies reflects the role of p53 loss in oligodendrocyte lineage cells or whether it is secondary to p53 loss in other cell types (i.e., neurons, astrocyte and microglia).

In in vitro experiments, p53 participates in thyroid hormone-induced oligodendrocyte progenitor cells (OPCs) differentiation [9]. It has been reported that high frequency of developmental abnormalities, including neuronal tube abnormalities and craniofacial malformations, have been observed in p53 knockout embryos [10,11]; however, whether this is related to deficits in oligodendrocyte development is not known. Therefore, the question of whether p53 plays a pivotal role in OL development in vivo still needs to be explored. By utilizing mice, in which Cre recombinase was expressed in 2′3′-cyclic nucleotide 3′-phosphodiesterase (*CNP*) gene locus starting at ~E12-E14 in CNS [12], we were able to disrupt a floxed TRP53 allele within OL lineage. In this study, we show that mice lacking p53 function in the OLs display normal phenotype under physiological conditions. The OL differentiation and myelin formation are normal in these animals histologically. To determine the role of oligodendroglia p53 in response to various stresses, three common mouse models of myelin damage were used: experimental autoimmune encephalomyelitis (EAE), cuprizone intoxication and white matter stroke.

## 2. Results

### 2.1. p53 Loss of Function in CNP+ Cell Lineage Does Not Affect Normal Oligodendrocyte Differentiation and Myelination

**Figure 1:**
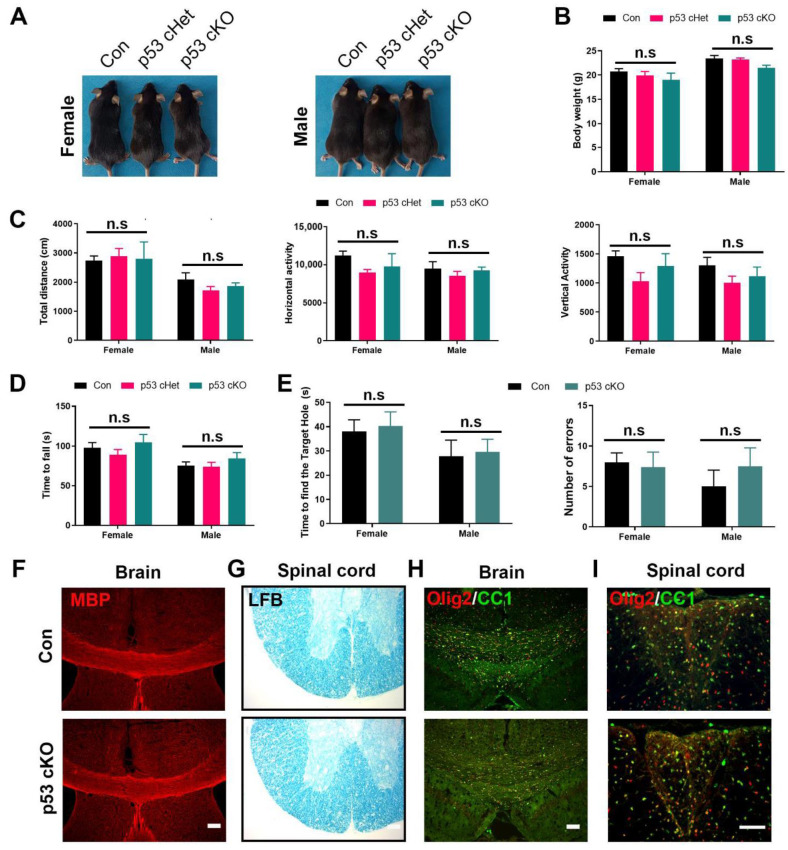
Behavioral and histological characterization of OL p53 cKO mice. Both female and male mice with different genotypes (control (Con), p53 cHet and p53 cKO) were examined. Body size (**A**) and body weight measurements (**B**) reveal no significant difference among genotypes at 2 months of age. There were no significant differences among genotypes in the open-field test for total distance, horizontal activity and vertical activity (**C**). Time to fall from rod in the rotarod test was not different among genotypes (**D**). There was no significant difference between genotypes in latency and number of errors to reach the target hole in the Barnes maze test (**E**). Representative immunohistochemistry images of MBP (**F**) showed no difference in the size of corpus callosum between Con and p53 cKO male mice. Representative Luxol fast blue (LFB)-stained sections (**G**) showed no difference in the size of spinal cord between Con and p53 cKO male mice. Representative immunohistochemistry images of olig2 and CC1 showed no difference in the number of OLs of corpus callosum (**H**) and spinal cord (**I**) between Con and p53 cKO male mice. (**B**–**D**): *n* = 7 female mice or 9 male mice for Con, *n* = 11 female mice or 12 male mice for p53 cHet, *n* = 6 female mice and 8 male mice for p53 cKO, one-way ANOVA test. The data are presented as mean ± sem. n.s: no significance. (**F**–**I**): Scale bar = 100 μm.

To investigate the effects of p53 in OL differentiation and myelination, we generated TRP53^flox/flox^; CNP^cre/+^ mice (p53 cKO), in which p53 is conditionally deleted by Cre recombinase specifically in CNP+ oligodendrocyte progenitor cells (OPCs) and mature OLs [13]. We observed that TRP53^flox/+^; CNP^cre/+^ mice (p53 cHet) and p53 cKO mice developed normally and did not display any obvious abnormalities in adult life. As shown in Figure 1A,B, p53 cHet and p53 cKO mice had comparable body size and weight at 2 months of age as compared to control mice (TRP53^flox/flox^ mice). The locomotor activity, motor coordination and spatial memory functions in p53 cHet and p53 cKO mice were also not altered (Figure 1C–E). Comparable myelin formation in the brain and spinal cords of control p53 cHet and p53 cKO mice was verified with MBP staining and LFB myelin staining at 2 months of age (Figure 1F,G). Furthermore, the results of immunofluorescence staining showed no alteration in the number of olig2+ and cc1+ cells in corpus callosum and dorsal spinal cords of p53 cHet and p53 cKO mice compared with control mice in the postnatal 2 months (Figure 1H,I). These data suggest that OL-specific p53 deletion did not significantly affect oligodendrocyte differentiation and myelin formation.

### 2.2. OL-Specific Loss of p53 Does Not Affect the Development of EAE

**Figure 2:**
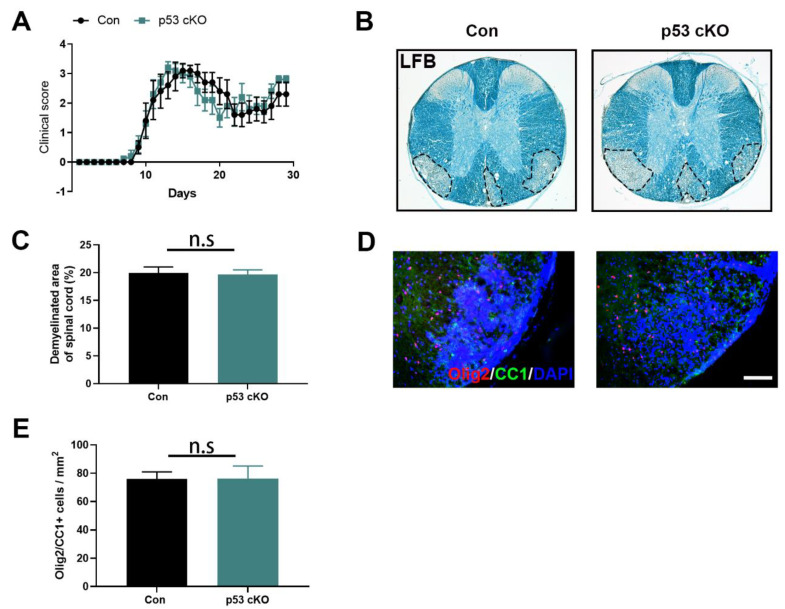
p53 deletion in OLs did not alter EAE severity. (**A**). Clinical score measurements showed no difference in the development of EAE and disease severity between Con and p53 cKO mice. (**B**,**C**). Luxol fast blue (LFB) staining of myelin showed no difference in the demyelination lesions at 30 days post EAE induction between Con and p53 cKO mice. Dashed lines demarcate lesion areas. (**D**,**E**). Representative immunohistochemistry images of olig2 and CC1 and their quantification of Olig2/CC1+ cells showed no difference in the number of OLs in the spinal cord at 30 days post EAE induction between Con and p53 cKO mice. (**A**): *n* = 6 mice for Con, *n* = 6 mice for p53 cKO, two-way ANOVA test; (**C**,**E**): *n* = 3 mice/group, two-tailed unpaired Student’s *t*-test. The data are presented as mean ± sem. n.s: no significance. (**D**): Scale bar = 100 μm. (**B**): Dotted lines demarcate lesion areas.

Since p53 is an important mediator in cell death in response to various stresses (i.e., inflammation and oxidative stress), we further studied the possible function of p53 in CNS demyelination. Firstly, we determined whether OL-specific p53 loss could provide protection against inflammatory CNS demyelination in EAE model, the most commonly used experimental model for multiple sclerosis. As shown in Figure 2A, control mice (TRP53^flox/flox^ mice) exhibited a typical EAE disease course. p53 cKO mice displayed a comparable disease course as compared to control mice. Quantitative analysis of LFB myelin staining showed that the percentage of area in the white matter of the lumbar spinal cord that was demyelinated in p53 cKO mice was comparable with control mice (Figure 2B,C). Similarly, olig2 and CC1 double immunostaining results indicated that p53 loss specifically in OLs did not significantly alter the number of oligodendrocyte lineage in the demyelinated lesions of EAE mice (Figure 2D,E). Taken together, these data suggest that loss of p53 function in cells of the OPC/OL lineage does not influence EAE disease severity and has no effect on oligodendrocyte loss and demyelination in the spinal cord of EAE mice.

### 2.3. p53 Deletion Protects against Cuprizone-Induced OL Damage

**Figure 3:**
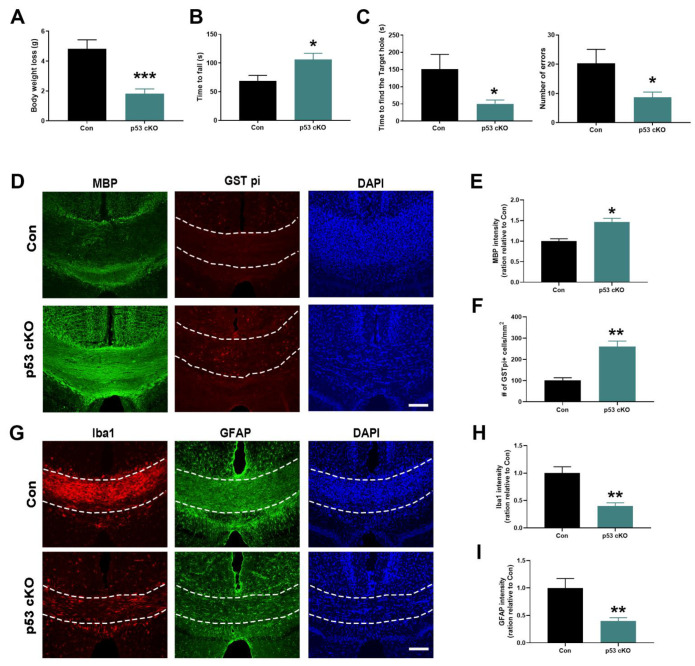
OL-specific p53-deficient (p53 cKO) mice are less susceptible to cuprizone-induced demyelination. Control (Con) and p35 cKO mice were examined after a 5-week cuprizone treatment. (**A**). The body weight loss was reduced in p53 cKO mice. (**B**). The time to fall from rod in the rotarod test was higher in p53 cKO mice than in Con mice. (**C**). The latency and number of errors to reach the target hole in the Barnes maze test were decreased in p53 cKO mice. (**D**). Representative immunohistochemistry images of MBP and GSP-pi in the corpus callosum of Con and p53 cKO mice after a 5-week cuprizone treatment. (**E**). Quantitative graphs of MBP intensity showed a significantly reduced myelin loss in p53 cKO mice compared with Con mice. (**F**). Quantitative graphs of GST-pi positive cell number showed more oligodendrocytes in the corpus callosum of p53 cKO mice compared with Con mice. (**G**). Representative immunohistochemistry images of GFAP and Iba1 in the corpus callosum of Con and p53 cKO mice after a 5-week cuprizone treatment. (**H**,**I**). Quantitative graphs of GFAP and Iba1 intensity showed a significantly reduced response of astrocyte and microglia to cuprizone intoxication in the corpus callosum of p53 cKO mice compared with Con mice. (**A**–**C**): *n* = 6 mice/group, two-tailed unpaired Student’s *t*-test; (**E**,**F**,**H**,**I**): *n* = 3 mice/group, two-tailed unpaired Student’s *t*-test. The data are presented as mean ± sem. * *p* < 0.05, ** *p* < 0.01, *** *p* < 0.001. (**D**,**G**): Scale bar = 100 μm. Dotted lines demarcate corpus callosum.

Since cuprizone can preferentially cause damage to mature oligodendrocytes in corpus callosum and other brain regions through inducing mitochondrial dysfunction, and p53 has a direct apoptogenic role at mitochondria through interacting with Bcl-xL and Bcl2 to induce mitochondrial outer membrane permeabilization [14], we asked whether p53 plays a role in oligodendroglia cell death in this demyelination model. As shown in Figure 3A, there was less body weight loss in p53 cKO mice after a 5-week cuprizone treatment, compared with control mice (TRP53^flox/flox^ mice). The OL-specific deletion of p53 gene significantly protected against the impairment of motor performance on the rotarod in p53 cKO mice (Figure 3B). Furthermore, spatial memory is more significantly preserved in cuprizone-challenged p53 cKO mice than that in control mice (Figure 3C). These protective effects were closely correlated with histological findings. MBP immunostaining was denser in p53 cKO animals compared with control animals after cuprizone intoxication (Figure 3D,E). The results of GST pi (a marker for mature OLs) staining showed that the number of OLs in corpus callosum were significantly higher in p53 cKO animals after a 5-week cuprizone treatment (Figure 3D,F). Consistent with the reduced demyelination, p53 deletion attenuated the activation of astrocytes (GFAP+ cells) and microglia (Iba1+ cells) in the corpus callosum of cuprizone-challenged p53 cKO mice (Figure 3G–I). These results indicate that p53 deletion can provide protective effects for OLs and myelin against cuprizone intoxication in brain.

### 2.4. p53 Deletion Alleviates White Matter Injury in White Matter Stroke

**Figure 4:**
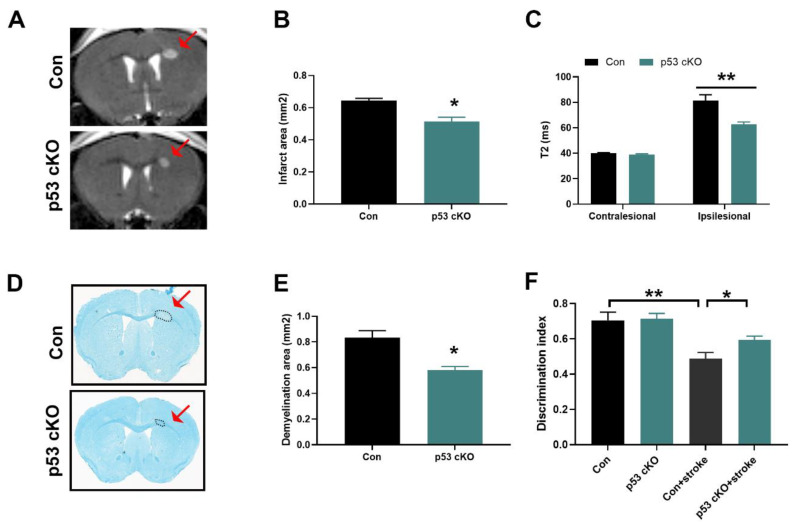
OL-specific loss of p53 function reduced brain injury and functional deficits after white matter stroke. (**A**). Representative T2-weighted MRI 3 days post stroke. (**B**). Quantitative graphs of T2-weighted MRI showed a significantly smaller lesion size in p53 cKO mice compared with Con mice. (**C**). Quantitative graphs of T2 relaxation time showed reduced edema in p53 cKO mice compared with Con mice. (**D**,**E**). Luxol fast blue (LFB) analysis showed reduced ischemic demyelination lesions in p53 cKO mice after 7 days compared to control mice. Dashed lines demarcate lesion areas. (**F**). p53 cKO mice showed a higher discrimination index than Con mice after white matter stroke in novel object recognition test. (**B**,**C**,**F**): *n* = 6 mice/group, two-tailed unpaired Student’s *t*-test for (**B**,**C**), one-way ANOVA test for (**F**); (**D**): *n* = 3 mice/group, two-tailed unpaired Student’s *t*-test. The data are presented as mean ± sem. The data are presented as mean ± sem. * *p* < 0.05, ** *p* < 0.01. (**A**,**D**): Red arrow and dotted lines demarcate lesion areas.

Our results above suggest that p53 plays different roles in the brain and spinal cord in response to various stresses. To confirm this hypothesis, we further examined the effects of p53 on oligodendrocyte survival in subcortical white matter stroke model. In this study, we carried out MRI scans to examine the edema and infarct volume after white matter stroke. Compared with control mice, MRI lesion volume was significantly smaller in p53 cKO mice on day 3 after L-NIO injection into corpus callosum (Figure 4A,B). Notably, there was also decreased T2 relaxation time in p53 cKO mice compared to control mice (Figure 4C), suggesting that brain edema was significantly lower in p53 cKO mice. To find out whether the early beneficial effect of OL-specific p53 deletion persisted at later time points, the brain tissue sections were examined 7 days after stroke using LFB myelin staining. p53 cKO mice showed decreased demyelination compared with control mice (Figure 4D,E). Young adult mice recovered motor control quickly, within weeks of the white matter stroke, and there was no difference in sensorimotor performances using Cylinder and grid-walk tests; however, they had prolonged cognitive deficit even 28 days after stroke when tested using a novel object recognition task [15,16]. Therefore, we determined whether OL-specific p53 loss protects against ischemic stroke-induced behavioral deficit using the novel object recognition test. As shown in Figure 4F, L-NIO injection to corpus callosum impaired the discrimination of the novel object 7 days after stroke in control mice. In contrast, p53 cKO mice following L-NIO injection had a significantly greater discrimination index than control mice. These findings suggest that p53 deletion in OL can provide protective effects against white matter stroke-induced damage.

## 3. Discussion

### 3.1. p53 Is Dispensable for Normal Oligodendrocyte Differentiation and Myelin Formation

Previous studies have shown that a fraction of p53-deficient embryos do not develop normally due to defects in neural tube closure [10,11]. For the survival of adult p53-deficient mice, a lack of p53 expression induces apoptotic brain lesions, learning deficiency and behavioral alterations, suggesting that p53 plays a crucial role in maintaining the integrity of the central nervous system [17]. However, it is still unknown what type of cells with p53 deficiency contribute to these behavioral deficiencies during development. OLs are major glial cells that ensheath axons in the CNS to ensure rapid saltatory conduction and provide metabolic support to neurons. p53 has shown to mediate normal oligodendrocyte development in vitro [9]. However, we report here that p53 was not required for oligodendrocyte differentiation and myelination in TRP53^flox/flox^; CNP^cre/+^ mice, in which Cre is induced just as OPCs begin to differentiate into OLs [18]. There were also no behavioral alterations in OL-specific p53 loss mice. During development, we also did not see any difference in neuronal numbers and locomotor activity in TRP53 ^loxP/loxP^; Camkinase II^Cre/+^ mice (p53 loss in forebrain neurons) and TRP53^flox/flox^; Slc6a3^Cre/+^ mice (p53 loss in dopaminergic neurons) [3,4]. These suggest that the function of p53 during the later stage of differentiation might not be important during development. However, as p53 plays a multifaceted role in cellular process and CNS-restricted transcription factor Olig2 affects a key posttranslational modification of p53 in both normal and malignant neural progenitors [19], whether p53 affects cell fate determination, OPC recruitment and proliferation deserves investigation in future studies.

### 3.2. The Important Role of p53 in the Stress-Induced OLs/Myelin Damage

Activation of p53 by cellular stress, genotoxic damage or inappropriate mitogenic cues results in growth arrest or apoptosis through transcription-dependent or mitochondria-dependent pathway. The functional significance of p53 for various stress-induced neuronal deaths has been largely established. In addition, p53 activities in glial cells contribute to various forms of neurodegeneration in a non-cell-autonomous fashion [20]. p53 deficiency may affect the activation and proliferation of astrocytes and microglia during kainic acid treatment [21]. p53 is persistently induced and contributes to human OLs apoptosis after tumor necrosis factor-alpha (TNFa) treatment in vitro [6]. Moreover, an in situ analysis of active MS lesions revealed increased p53 expression in OLs, and p53 overexpression can cause human OLs apoptosis [22]. p53 has also been shown to be activated in OLs in response to HIV infection [23]. However, the in vivo experimental data about the role of p53 in OL under stress conditions is less studied.

In the cuprizone model of demyelination, genetic deletion or pharmacological inhibition of p53 reduced microglial activation and myelin loss in the corpus callosum of mice [8]. However, whether these effects are due to a direct role of p53 in OL lineage cells or an indirect role of p53 in other cell types is not known. In our study using OL-specific p53 cKO, we found that mice with OL-specific p53 loss had reduced demyelination compared with that in wild-type mice in response to cuprizone feeding and L-NIO-induced white matter stroke. These results indicate the essential role of p53-mediated signaling in cuprizone or ischemic-induced demyelination. Conversely, p53-deficient mice displayed more extensive demyelination and a more severe disease course in MOG_35-55_ EAE model compared with wild-type mice [24]. In the present study, we found that mice with OL-specific p53 loss displayed a comparable disease course and demyelination as compared to control mice, suggesting that p53-mediated signaling may not be involved in OL damage in EAE-induced demyelination model.

The differential role of p53 in these three demyelination models might be due to the OL heterogeneity between spinal cord and brain, the gender differences and different cellular stresses. The MOG_35-55_-induced EAE is a T-cell-mediated autoimmune disease of the CNS that causes the myelin mainly in spinal cord to be recognized as an exogenous immunogen and subsequently be attacked by T-cells [25]. Female mice are commonly used to construct EAE model and have a higher neurological motor impairment than male mice in MOG_35-55_ EAE model [26]. However, cuprizone selectively affects mature oligodendrocytes in the brain, and oxidative stress is a direct cause of apoptosis [27,28]. L-NIO is a potent non-selective inhibitor of nitric oxide synthase (NOS) and can induce local ischemia at the site of injection, resulting in oxidative stress and inflammation [29]. Moreover, single-cell transcriptomic has revealed OL heterogeneity in mouse and human CNS [30,31]. Therefore, further studies are needed to investigate whether p53 is upregulated or activated (nuclear translocation or mitochondrial translocation) in these different models in OL.

In conclusion, our findings underscore the important role that p53 plays following cuprizone or white matter stroke-induced myelin damage. We propose that modulation of p53 can be a therapeutic target for ischemic white matter disease and multiple sclerosis. However, the exact molecular mechanisms through which p53 mediates OLs/myelin damage, especially through transcriptional modulation or mitochondrial pathways, are not known and warrant further investigation in future studies.

## 4. Materials and Methods

### 4.1. Animal Study

All animal experiments followed the guidelines of the National Institutes of Health (NIH, New York, NY, USA), and the animal protocols were approved by the Institutional Animal Care and Use Committees (IACUC) of Case Western Reserve University and University of Cincinnati. The mice were housed in a temperature-and humidity-controlled animal facility with a 12 h light/dark cycle and food and water available ad libitum. TRP53^flox/flox^ mice [32] and CNP-cre mice obtained from Dr. K.A. Nave [13] were on C57BL/6J background. To generate a conditional knockout of p53 in oligodendrocytes, we firstly crossed TRP53^flox/flox^ mice with Heterozygous CNP-cre mice to generate TRP53^flox/+^; CNP^Cre/+^ mice, which were then crossed to TRP53^flox/flox^ mice to produce conditional knockout mice of TRP53^flox/flox^; CNP^Cre/+^ (p53 cKO). Genotypes were determined by PCR from the DNA extracted from tail tips. TRP53 floxed alleles were genotyped by the primer set (5′-CACAAAAACAGGTTAAACCCAG-3′; 5′-AGCACATAGGAGGCAGAGAC-3′). CNP-cre gene was genotyped using primers (5′-GCCTTCAAACTGTCCATCTC-3′; 5′-CCCAGCCCTTTTATTACCAC-3′; 5′-CATAGCCTGAAGAACGAGA-3′). Both females and males were used in this study. TRP53^flox/flox^ mice served as controls for this study.

### 4.2. Experimental Autoimmune Encephalomyelitis (EAE)

MOG_35-55_-induced EAE is a common animal model for MS, which is characterized by paralysis, inflammation and demyelination. It is mediated by myelin-specific CD4+ T cells. MOG_35-55_-induced EAE model was performed as previously described [33,34]. The p53^flox/flox^ and p53 cKO mice (female, 10 weeks old) were immunized with MOG_35-55_ together with complete Freund’s adjuvant emulsion (Hooke Laboratories, MOG_35-55_ EAE Induction kit, EK-2110) according to the manufacturers’ instruction. Briefly, the mice were immunized via subcutaneous injection of 200 μL of MOG35-55 peptide in complete Freund’s adjuvant. Pertussis toxin (250 ng) was injected intraperitoneally at 2 h and 24 h post immunization. All EAE mice were monitored daily and scored using a clinical scale from 0 to 5 (0: no abnormality; 1: limp tail; 2: limp tail and hind leg weakness; 3: limp tail and complete paralysis of hind legs; 4: hind leg and partial front leg paralysis; 5: moribund).

### 4.3. Cuprizone-Induced Demyelination Model

Cuprizone is a copper-chelating mitochondrial toxin that causes oligodendroglial cell death and demyelination by an inhibition of complex IV of the mitochondrial respiratory chain and inducing megamitochondria in the mouse brain [35]. To induce demyelination in the brain, the p53^flox/flox^ and p53 cKO mice (male, 10 weeks old) were fed with a standard 0.3% cuprizone diet in chow (Harlan Laboratories) for a period of 5 weeks. The body weight of all mice was monitored once a week. All mice were assessed for motor and cognitive deficits using open-field activity test, rotarod test and Barnes maze test in a double-blinded manner.

### 4.4. White Matter Stroke

Local injection of an irreversible eNOS inhibitor, N5-(1-Iminoethyl)-L-ornithine HCl (L-NIO), has been shown to be able to reliably produce a focal stroke in murine white matter with no known paracrine effects on cellular elements of white matter [36,37]. White matter stroke was performed as described with minor modifications [38]. Briefly, L-NIO (27 mg/mL in sterile physiological saline; EMD/Millipore) was microinjected into subcortical white matter via micropipette at an angle of 36° in the p53^flox/flox^ and p53 cKO mice (male, 10 weeks old). Three stereotaxic injections (each consisting of 200 nL of L-NIO solution) were made in the following coordinates: the first injection at anteroposterior (AP) +0.22 mm, mediolateral (ML) +0.22 mm, dorsoventral (DV) −2.10 mm; the second injection at AP+0.70 mm, ML+0.15 mm, DV−2.16 mm; and the third injection at AP+1.21 mm, ML+0.15 mm, DV −2.18 mm.

### 4.5. Magnetic Resonance Imaging (MRI)

The mice were subjected to imaging session 1 day after white matter stroke by staff that were blinded to the genotypes/treatment groups. MRI studies were performed on a vertical wide bore 9.4T Bruker Avance III HD scanner with a 36 mm proton volume coil. T_2_-weighted anatomical coronal images of the brain were acquired with a fat suppressed 2D rapid acquisition with relaxation enhancement (RARE) sequence using the following parameters: TR 4 s, TE 71.5 ms, echo spacing 6.5 ms, 9 slices, slice thickness/gap 0.75/0.3 mm, RARE factor 20, receiver bandwidth 67 k, averages 4, matrix 192 × 192, FOV 28.4 × 28.4 mm and total scan time 2.24 min. In addition, multi-slice multi-echo (MSME) spin echo images with 20 echo times varying from 9.21 to 184.14 ms were used to calculate T_2_ relaxation time maps by fitting the image data to a monoexponential decay curve within Bruker’s Paravision 6.0.1 software. Other MSME acquisition parameters included: TR 2 s; echo spacing 9.207 ms, 5 slices, slice thickness/gap 0.75/0.75 mm, receiver bandwidth 50 k, averages 2, matrix 142 × 142, FOV 28.4 × 28.4 and total scan time 7 min.

### 4.6. Behavioral Test

#### 4.6.1. Open-Field Activity Test

General locomotion activity in mice was assessed using automated open-field Accuscan activity monitors (Columbus, OH, USA). There were 16 horizontal and 8 vertical infrared sensors (interval 2.5 cm) in each chamber. Each mouse was put into a 42 × 42 × 31 cm Plexiglas open box for 1 h with food and water supply. To avoid observer bias, this locomotor test was automatically monitored by the computer and software. Locomotor activity was calculated by automated Fusion software (Accuscan, Columbus, OH, USA). The following variables were measured: (A) horizontal activity (the total number of beam interruptions that occurred in the horizontal sensors); (B) total distance traveled (the distance traveled by the animals in cm); (C) vertical activity (the total number of beam interruptions that occurred in vertical sensors).

#### 4.6.2. Rotarod Test

Motor coordination and balance of the mice were evaluated using an accelerating rotarod test. A rotarod apparatus (Accuscan Instruments, Columbus, OH, USA) with a 30-mm-diameter rod was used in this study. On training day, the mice were placed in separate lanes on the rod rotating at 5 rpm. After 60 s on the rod, the animals were returned to the home cage. The procedure was repeated for a total of 3 trials separated by 15 min intertrial intervals. On testing day, the mice were placed on the rod apparatus rotating from 4 to 40 rpm over 5 min. After falling off the rod, the mice were immediately placed back in their home cages. Latency to fall and the distance traveled on the rotarod before falling were recorded. The test was stopped after a maximum of 5 min. This procedure was repeated 2 more times with 15 min intertrial intervals. The apparatus was cleaned with 75% ethanol (*w*/*v*) after each trial. The averages of the 3 trials were used for statistical analyses.

#### 4.6.3. Barnes Maze Test

The spatial memory of mice was examined using Barnes maze (Stoelting Company, Wood Dale, IL, USA). The maze consisted of a 91.5 cm diameter circular platform with 20 holes around the perimeter. Blowing fans and a bright light above the platform were used to encourage mice to find the escape tunnel placed under the target hole. On day 0, the mice were placed in a start chamber in the middle of the platform. After 10 s, the start chamber was lifted and the mice were gently guided to enter the target. On day 1, the mice were trained at 4 trials in 2 sessions to find the escape tunnel. Once the mice entered the target hole, the hole was covered, and the mice were allowed to stay in it for 2 min. If the mice could not locate the target hole within 5 min, they were guided by the observer to enter the target hole. On day 2, one trial was run and video-taped until the mice entered the target hole or stopped after 5 min if the mice could not locate the target hole. The time spent to locate the escape hole and the number of errors made by the mice in finding the hiding hole were measured by an observer blinded to the animal treatment group.

#### 4.6.4. Novel Object Recognition Test

The mice were placed individually in a rectangular arena (35 cm × 20 cm). During habituation, the mice were allowed to explore an empty arena for 5 min. Twenty-four hours after habituation, the mice were exposed to two identical objects (green cylinder, familiar objects). The next day, the mice were exposed for 5 min to a familiar object and a novel object (blue rectangular box). The time spent exploring each object and the discrimination index percentage were calculated.

### 4.7. Immunohistochemistry

The mice were anesthetized and perfused transcardially with PBS and 4% PFA. The spinal cord and brain were dissected and postfixed in 4% paraformaldehyde (PFA) overnight at 4 °C, followed by equilibration in 20% sucrose and 30% sucrose. The 20 μm cryosections were collected and stored at −80 °C. Antigen retrieval was performed using Reveal Decloaker solution (Biocare Medical, Pacheco, CA, USA) or citrate buffer before immunostaining. The sections were incubated with primary antibodies overnight at 4 °C (MBP: SMI-99, 1:500, Covance; Olig2: 1:250, Millipore, Burlington, MA, USA; CC1: 1:250, Calbiochem, San Diego, CA, USA; Iba1: 1:500, Wako Chemicals, Richmond, VA, USA; GFAP: 1:500, DAKO, Glostrup Kommune, Danmark; GST-pi: 1:250, Abcam, Cambridge, UK), washed and incubated with fluorescently conjugated secondary goat anti-rabbit IgG AlexaFluor-594 or goat anti-mouse IgG 488 (1:500; Invitrogen, Waltham, MA, USA) antibodies for 1 h at room temperature. Images were collected and analyzed using a Leica fluorescence microscope or Olympus fluorescent microscope (Olympus Inc., CenterValley, PA, USA).

### 4.8. Luxol Fast Blue (LFB) Myelin Staining and Quantification

LFB staining was performed according to the manufacturer’s instructions (#26681, Electron Microscopy Sciences, Hatfield, PA, USA). The 20 μm tissue sections were incubated in LFB solution at 56 °C overnight and then rinsed sequentially with 95% alcohol and distilled water. The sections were then placed in 0.1% lithium carbonate solution and dehydrated with a series of graduated ethanol, cleared with Histoclear and mounted. A set of serial matched sections were imaged and analyzed. Images (5 to 6 sections/animal) were captured under the light microscope. The demyelinated areas (lack of LFB staining) were quantified using ImageJ software. For EAE sections, demyelinated areas were measured and represented as a percentage of total area of spinal cord. For sections of the white matter stroke model, lesion volumes were calculated by the lesion area from serial sections throughout the entire lesion.

### 4.9. Statistical Analysis

Data were presented as mean ± SEM. Data statistical analysis was performed using the one-way or two-way ANOVA tests for comparison of various groups. Student’s *t*-test was performed for comparison between two groups. *p* values of < 0.05 were considered statistically significant.

## Data Availability

The datasets used and/or analyzed in the current study are available from the corresponding author on reasonable request.
